# Color Doppler ultrasound morphology of glomus tumors of the extremities

**DOI:** 10.1186/s40064-016-2883-0

**Published:** 2016-08-11

**Authors:** Zhina Fan, Gang Wu, Bing Ji, Cunfeng Wang, Shuaiwei Luo, Yinlong Liu, Jianjun Yuan

**Affiliations:** Department of Ultrasonography, Henan Provincial People’s Hospital, Zhengzhou, 450003 China

**Keywords:** Glomus tumors, Extremities, Color Doppler flow imaging, Diagnosis

## Abstract

**Objective:**

The aim of this study was to investigate the value of color Doppler flow imaging (CDFI) in the diagnosis of glomus tumors in the extremities.

**Methods:**

Sonography results of 62 nodules of 50 patients with glomus tumors in the extremities confirmed by surgery and pathology were analyzed retrospectively.

**Results:**

The sex ratio in the group of 50 patients was (female:male) = 5.25:1. Glomus tumors were more common in women aged 30–40 years. 84 % (42/50) of glomus tumors occurred in the fingers, with the thumb being the most common. 2D results showed that 64.52 % (40/62) of 62 nodules were hypoechoic, 30.65 % (19/62) were heterogeneous echo, 4.84 % (3/62) were hyperechoic; 64.52 % (40/62) had a clear border, while 35.48 % (22/62) had an unclear border. CDFI showed that 38.71 % (24/62) had visible rich blood in the tumor, 35.48 % (22/62) had little visible blood in the tumor, and 25.81 % (16/62) had no significant intratumoral blood flow. Of the 50 patients, 92 % (46/50) showed a fixed contact pain, were sensitive to cold stimuli, which was improved by hot water and air, and for which anti-inflammatory treatment was ineffective.

**Conclusion:**

Glomus tumors in the extremities had certain ultrasound features, and its internal blood flow was diverse. Understanding this feature may be helpful for the diagnosis of non-typical glomus tumors.

## Background

Glomus tumor was a rare benign soft tissue, originated in systemic fine glomus in arteriovenous anastomoses, which can occur in the limbs, trunk, neck, and internal organs; it was more common in the limbs (Dalrymple et al. 1997). Owing to the low incidence, inadequate clinical knowledge, and high misdiagnosis rate (Sun et al. 1996), some scholars believed that magnetic resonance imaging (MRI) had a distinct advantage in the diagnosis of glomus tumors; however, because of its high cost, it was difficult to promote (Jablon et al. 1990; Dalrymple et al. 1997; Al-Qattan et al. 2005; Takemura et al. 2006). With the improvement of resolution, high–frequency ultrasound can not only clearly show the characteristics of the tumor in real time, but can also accurately locate the position, which was more widely used in the preoperative examination of glomus tumors. Previous literature (Chen et al. 2003; Matsunaga et al. 2007; Park et al. 2011) reported that typical sonographic features of most glomus tumors were soft-tissue tumors with clear boundaries and visible signals of internal rich blood flow. In this study, we mainly observed the blood distribution inside the tumor by summarizing sonographic features of glomus tumors.

## Methods

Preoperative color Doppler flow imaging (CDFI) of 62 nodules of 50 patients with glomus tumors in the extremities confirmed by surgery and pathology during January 2012 and October 2014 were analyzed retrospectively to discuss the diagnostic value of CDFI in glomus tumors in the extremities.

### Study subjects

There were 50 patients in this study group, including 8 males and 42 females, with a sex ratio of female to male = 5.25:1, aged 18–86 years with a mean age of (43.00 ± 15.26) years. These cases were treated due to local pain or unintentional palpable or mass; 47 cases were single, and 3 cases were multiple. The tumor locations are shown in Table 1. Twenty-two cases had tumors under the fingernails, 13 cases with tumors next to the nail, 6 cases had tumors in pulp, 1 case in finger-proximal palmar, 4 cases in calves, 2 cases in ankles, and 2 cases in toes. This study was conducted in accordance with the declaration of Helsinki. This study was conducted with approval from the Ethics Committee of Henan Provincial People’s Hospital. Written informed consent was obtained from all participants.

**Table 1:** Distribution of 50 cases with glomus tumor

Location	Cases	Proportion (%)
Under fingernails	22	44
Next to the nail	13	26
Pulp	6	12
Finger proximal palmar	1	2
Calf	4	8
Ankle	2	4
Toe	2	4

### Methods

Acuson S2000 color Doppler ultrasonography (Siemens AG, Germany), Hitachi HI Vision 900 ultrasound diagnostic (Hitachi, Tokyo, Japan) with probe frequencies of 6.0–13.0 MHz were used. The probe was placed on the pain or mass parts of the patients for scanning. An optimal image was obtained by adjusting the depth, gain, and filtering based on the specific location of the lesion. After finding the tumor, 2D ultrasound was used to observe the tumor boundary, capsule, shape, internal echo, and its relationship with the surrounding tissues, and the size of the tumor was measured. Low-flow color Doppler settings were used to permit optimal visualization of small vessels. The blood flow within the tumor was observed by CDFI. The quantification of blood flow inside the tumor was classified according to the Adler semiquantitative method (Dottt et al. 1990): low flow is defined as occurring when the signal shows flashing, unstable, dotted blood flow, and rich blood flow is defined if the signal shows sheet-like, striped, or dendritic blood flow. Pulsed Doppler sampling was used for spectral measurement.

## Results

### 2-D and color Doppler ultrasonography

**Fig. 1:**
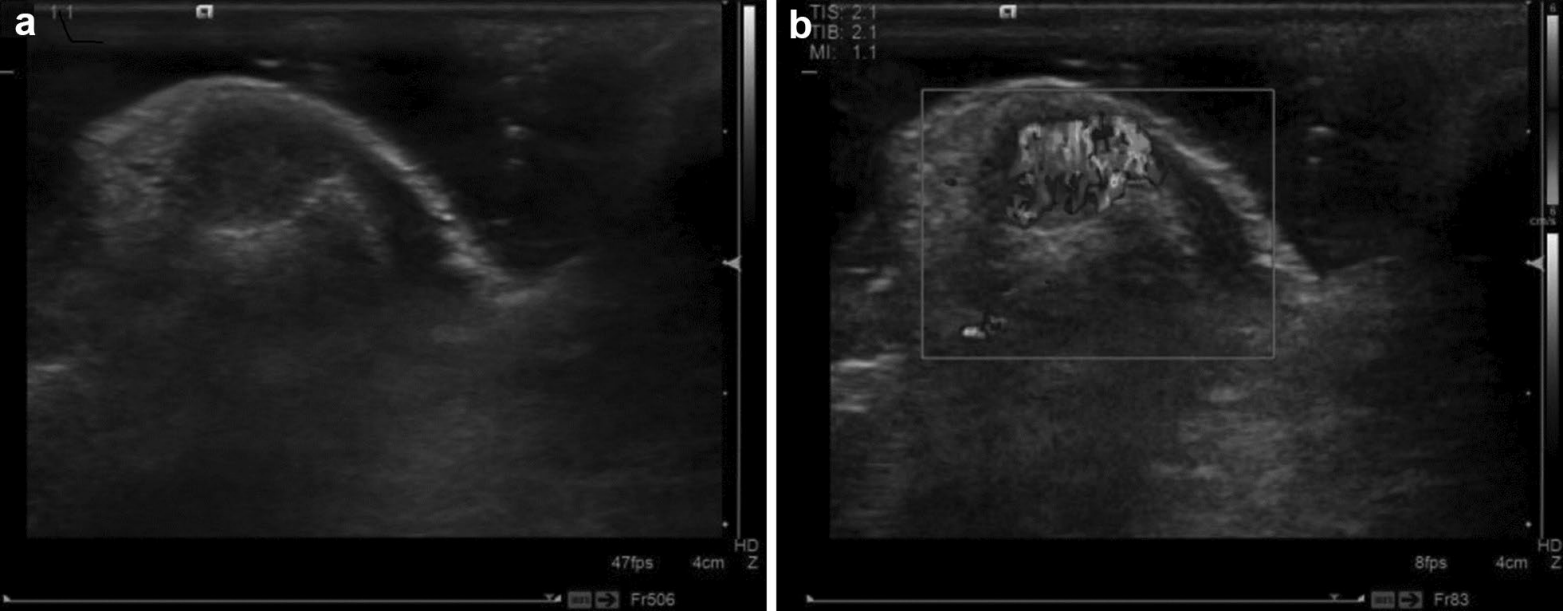
Ultrasound images of female patient (27 years old). **a** Two-dimensional ultrasound showed hypoechoic partial ulnar side in right thumb, with clear border and regular shape, the rear phalanx pressed to be curved; **b** CDFI showed rich blood flow in tumor tissues

**Fig. 2:**
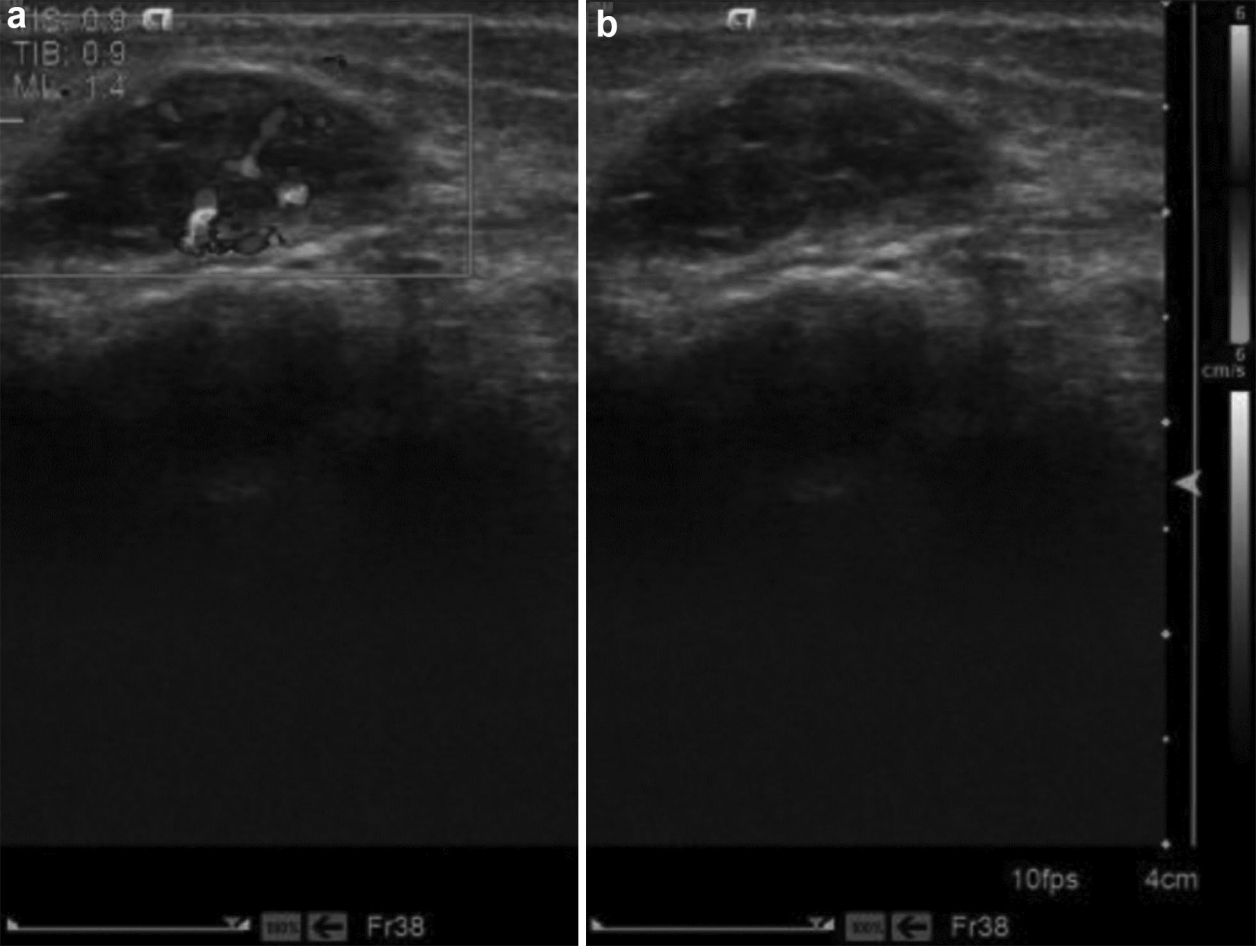
Ultrasound images of female patient (18 years old). **a** CDFI showed a little intratumoral blood flow signal; **b** Female, 18 years old, hypoechoic inside the subcutaneous bundle of the right side for lower leg, and muscle gap of the right foot, the figure showed that a little blood flow signal was visible in the muscle bundle of the middle of the calf with clear border

**Fig. 3:**
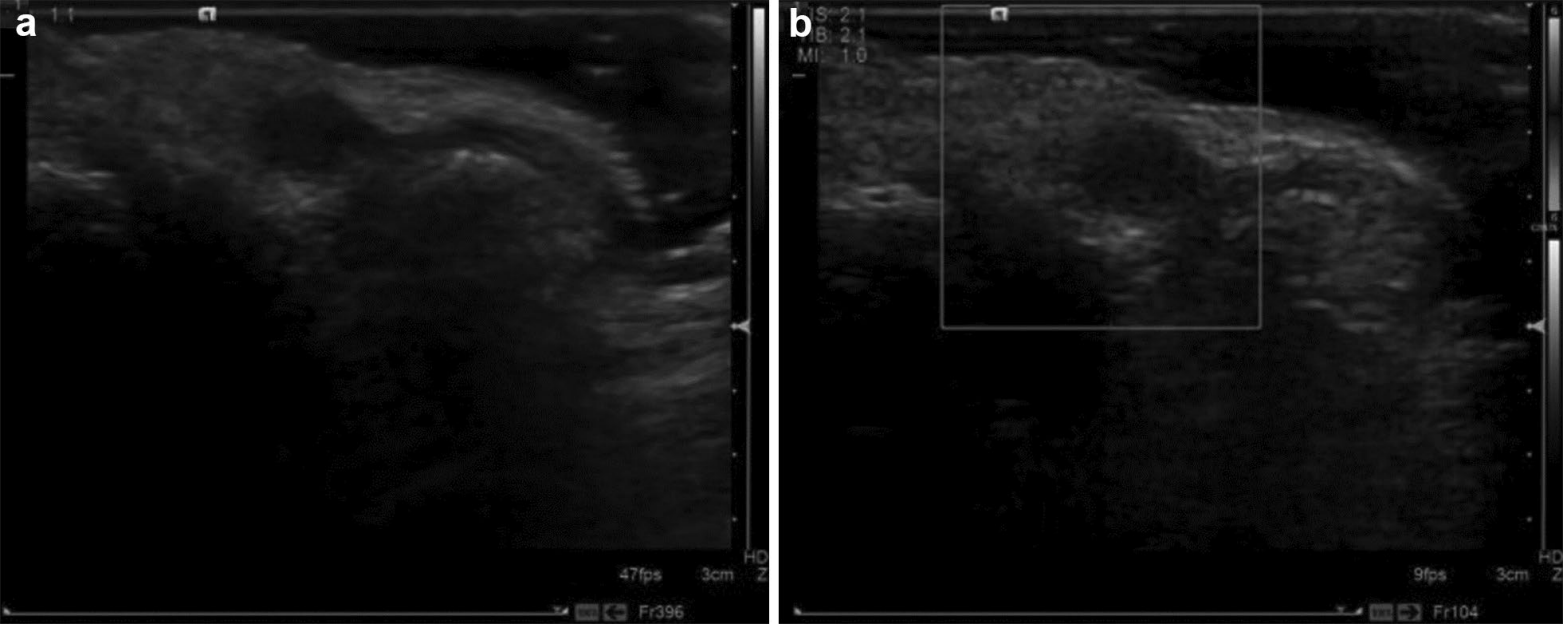
Ultrasound images of female patient (45 years old). **a** Two-dimensional ultrasound showed hypoechoic in the inside subcutaneous of the second toenail root of the left foot with clear border; **b** CDFI showed no intratumoral blood flow. The *Figure* indicated that the node was located in subcutaneous tissue adjacent to root of hyponychium

In this study, the size of glomus tumor of in the cases considered was approximately 1.5–9.7 mm with an average of approximately (5.7 ± 1.9 mm). The results of color Doppler ultrasonography of glomus tumors are shown in Table 2. The classic ultrasonography of glomus tumors showed solitary hypoechoic lesions with clear boundaries and regular shape, and internal abundant flow signals were observed (Fig. 1). In this group of patients, 64.52 % showed clear tumor borders and regular shapes, while 35.48 % had blurred tumor boundaries. In 62 tumors, rich blood flow was visible in only 38.71 % of tumor tissues, while little blood flow in 35.48 % (Fig. 2), and no significant blood flow in 25.81 % (Fig. 3). In the subungual glomus tumors, 19.4 % showed cortical bone–adjacent pressure, while 80.6 % showed no abnormal adjacent cortical bone.

**Table 2:** Distribution of finger glomus tumors for 42 cases with glomus tumor on the hands

Location	Right hand (cases)	Left hand (cases)	Total proportion (%)
Thumb	10	11	50.0
Forefinger	2	4	14.3
Middle finger	4	1	11.9
Ring finger	3	1	9.5
Little finger	3	3	14.3

### Doppler ultrasonography

Blood flow signals were detected in 46 of 62 nodules, including 24 with rich blood flow, 22 with little blood flow, and 16 with no significant blood flow (Table 3). The peak systolic velocity (PSV) inside the nodules, end-diastolic velocity (EDV), and vascular resistance index (RI) were measured using pulsed Doppler. PSV ranged 4.9–25.6 cm/s, PDV ranged 1.5–11 cm/s, and RI ranged 0.46–0.71. Patients with RI > 0.6 accounted for 36.96 % (17/46).

**Table 3:** The diagnostic results of glomus tumors by Doppler ultrasonography of (n = 62)

Items	Internal echo			Border		Capsule		Blood flow			Cortical bone compression	
	Low	Mix	High	Clear	Unclear	Yes	No	Rich	A little	No	Yes	No
Cases	40	19	3	40	22	18	44	24	22	16	12	50
Proportion (%)	64.5	30.7	4.8	64.5	35.5	29.0	71.0	38.7	35.5	25.8	19.4	80.7

### Ultrasound diagnosis rate

Six out of 50 cases with glomus tumors in the extremities were misdiagnosed; the diagnosis rate was 88 %, of which, three cases were misdiagnosed, and the other three cases were located under the nail and toe.

All incisions after surgery were stage I healing. Postoperative pain symptoms completely disappeared. The follow-up lasted for 6 months to 3 years and 4 months with an average of 1 year and 2 months; no tumor recurrence occurred and physical functions were normal.

### Clinical presentations

Glomus tumor was more common in females aged 30–40 years; 56 % (28/50) showed blue subcutaneous nodules that were softly touched, 44 % (22/50) did not show significant changes; the skin temperature was normal, and part of the skin had no significant changes. Localized tenderness was the primary symptom, where 92 % (46/50) showed stable contacted pain, and showed more severe pain when it was cool, which improved when it was warm; anti-inflammatory treatment was ineffective.

## Discussion

Glomus tumors were relatively rare with unclear causes; some literature (Glazebrook et al. 2011) reported that it may be related to an injury. This group had no clear history of trauma; therefore, the incidence may be due to glomus hyperplasia. Glomus tumors can occur in various parts of the body, but the finger or toe end and subcutaneous tumors were more common. In this group, of the 50 cases with glomus tumor in the extremities, 44 cases involved the finger or toe, among which finger end tumors were the most common. Owing to the occult site, the disease was difficult to diagnose, X-ray results were negative manifestations, and the positive diagnosis rate was low. When glomus tumor volumes enlarged, X-ray showed bone cortex curved depressions. Twelve of 50 patients in this group had cortical bone compression, and thus, ultrasound suspected that tumor destroyed bone, which should be combined with X-ray examination, while routine X-ray examination was not recommended (Ying et al. 2009). There is currently a controversy regarding the diagnostic value of MRI for glomus tumors before surgery. Ham et al. (2013) reported that MRI had a good diagnostic value in glomus tumors. Studies of Al-Qattan et al. (2005) suggested that MRI had little diagnostic value in clinically diagnosed glomus tumors. In addition to being combined with venography, MRI was expensive, which also limited its wide range of application.

Studies of Chiang et al. (2014) showed that 92 % (11/12) of the tumor or margin had rich intratumoral blood flow signal. Wortsman and Jemec (2009) also reported that seven cases of glomus tumor showed hypoechoic lesions with clear borders, where internal rich blood flow signals were recorded. However, our series showed that only 38.71 % (24/62) of tumors had visible rich blood flow in tumor tissues, 35.48 % (22/62) had little visible blood in tumor tissues, and 25.81 % (16/62) had no intratumoral blood flow. A case report by Perks et al. (2003) reported that a juxtacortical glomus tumor of the distal femur had no color signal, possibly because of the deep nature of the lesion. According to the author’s analysis, this was possibly related to the deep position of glomus tumors as well as the difficulty to show blood flow. The major reason may be derived from other factors, including that the frequency of probe used is 13 MHz, while it was 15 MHz in Wortsman and Jemec’s study. Different setting conditions may further influence the accuracy. On the other hand, this may be related to the pathology of glomus tumors. Glomus tumors comprised of different proportions of glomus cells, vascular, and smooth muscle divided into classical glomus tumor, glomangioma, glomangiomyoma, and myxoid glomus tumor according to the proportion. The classical glomus tumor comprised rich blood vessels and flaky tumor cells around blood vessels in the observation under light microscopy; therefore, rich red and blue blood flow were visible inside in colored spherical shape, and low resistance blood flow spectra could be measured. This was the most common type, combined with a series of clinical symptoms of glomus tumors, positive diagnosis that could be made. Only little blood flow was generally visible in glomangioma, while ultrasound cannot detect blood flow in glomangiomyoma and myxoid glomus tumors due to rare vascular or vasodilatation with thrombosis. Thus, ultrasound of glomus tumors were characterized by diversity. Typical glomus tumors showed single low echo with a clear boundary and visible rich blood flow signals. The PSV inside the nodules measured in this study ranged from 4.9 to 25.6 cm/s, PDV ranged from 1.5 to 11 cm/s, and RI ranged from 0.46 to 0.71. RI > 0.6 accounted for 36.96 % (17/46), which was basically the same as the PSV in the range of 3.7–26.1 cm/s from the measurement of five cases with glomus tumors by Wortsman and Jemec (2009). Currently, only blood flow for cases with glomus tumors have been reported (Kree et al. 1986; Chisci et al. 2008); there have still been no specific reports about the internal hemodynamics of glomus tumors, the influence of which on surgical treatment and prognosis needs to be further studied. The diagnosis of atypical ultrasound glomus tumors should be combined with clinical manifestations and combined with MRI when necessary.

Ultrasound misdiagnosis reason analysis of glomus tumor in the 50 patients, three multiple cases were misdiagnosed due to the lack of knowledge of clinical characteristics of glomus tumors. Most glomus tumors were solitary, while approximately 25 % of the cases were multiple. Schiefer et al. (2006) (Anakwenze et al. 2008) found that this type of glomus tumor was more common in children, mainly in the lower limbs, especially knee and ankle joint surroundings. The other three cases were misdiagnosed because of no obvious pain to contact, no significant blood flow inside tumor, or only little visible blood flow. These included two cases where glomus tumors had no significant pain to contact, but were accompanied by cold stimuli-sensitivity, and one case with worsening pain following hot and sour food stimulation. Taking full advantage of real-time ultrasound and detailed communication with patients, diagnostic accuracy can be improved, for which MRI can be advantageous.

Notes on the diagnosis of glomus tumors: (1) For subungual glomus tumors with small size and hidden incidence, the operation should not be forced. Lightly spinning the probe and multi-coating coupling agent, putting a diagnostic pressure on the affected area to facilitate the discovery of small lesions, and performing contralateral compared scanning should be performed when necessary. (2) When viewing the internal tumor blood flow, no pressure should be applied, noting to reduce the scale, or use power Doppler to facilitate in displaying the internal blood flow. (3) Not all glomus tumors were rich in blood. Different pathological types had different internal blood flows. (4) For multiple lesions, especially in the lower limbs, knee, or ankle surroundings, the possibility of glomus tumors should be considered even without ultrasonic characteristics of typical glomangioma accompanied by intermittent pain, tenderness, and subcutaneous nodules sensitive to temperature changes (Tony et al. 2013).

Differential diagnosis of glomus tumor: (1) Tumor type: hemangioma: the disease usually occurred after birth or about 2 weeks after birth, showing scattered or flake erythema with local swelling and accompanied by subcutaneous tumor. Usually the tumor showed rapid development within 1 year. The sonographic glomus tumor and the true hemangioma were more difficult to identify, but the former resulted in pain from more pressure and often occurred in the finger, while the latter was more common in infants and young children. (2) Vascular malformations: ultrasound was more irregular, no pain to pressure, and no specific surface distribution. Glomus tumor ultrasound manifestations were regular and pain was the most important sign. (3) Rhabdomyosarcoma: it was more common in the quadriceps, but without significant change in surface color. (4) Melanoma: it had a high degree of malignancy and metastasis was rapid with a short duration; glomus tumors had slow progression, patients had a long history (Carlson et al. 2015).

Treatment of glomus tumors: surgical resection was the only effective treatment (Lee et al. 2013). Traditionally, local tumor excision was used, from which relapse was common. In new treatment methods, after using urea injection for sclerotherapy to gradually close blood vessels, tumor resection is performed after hardening it, which can reduce the relapse rate, and the suffering and economic burden of patients can be alleviated (Faisan Smilevitch et al. 2014). Preoperative color Doppler ultrasound can accurately locate the tumor and provide the size of the tumor, distance to superficial, borders, whether it is with or without capsule, and relationship between internal blood flow and the surrounding tissues, which are important parameters for surgery.

## Conclusion

In summary, in contrast with previous studies, we found that not all glomangioma had rich blood flow; little or no blood flow signal was visible in certain tumor tissues. For this case, the typical clinical presentation should be assisted for diagnosis to prevent omitting diagnosis or misdiagnosis. However, we did not perform the relevant statistical analysis due to the small sample size in this study.
